# Genome Sequence of Ex-Afghanistan Crimean-Congo Hemorrhagic Fever Virus SCT Strain, from an Imported United Kingdom Case in October 2012

**DOI:** 10.1128/genomeA.00161-13

**Published:** 2013-05-16

**Authors:** John Chamberlain, Barry Atkinson, Christopher H. Logue, Jennie Latham, Edmund N. C. Newman, Roger Hewson

**Affiliations:** Virology & Pathogenesis Group, Microbiology Services – Research, Health Protection Agency, Porton Down, Salisbury, Wiltshire, United Kingdom

## Abstract

Crimean-Congo hemorrhagic fever (CCHF) virus is a serious human pathogen causing severe hemorrhagic disease with a fatality rate of up to approximately 30%. We have determined the viral genomic sequence from an isolate that caused a fatal case of imported CCHF in the United Kingdom in October 2012.

## GENOME ANNOUNCEMENT

Crimean-Congo hemorrhagic fever virus (CCHFv) causes severe hemorrhagic fever with fatal outcome in approximately 30% of cases (1); it is also the most geographically widespread of the viral hemorrhagic fevers and medically important tick-borne diseases (2). This arbovirus, of the *Nairovirus* genus in the *Bunyaviridae* family, can be found across much of Asia, from China and the Indian subcontinent, to Eastern Europe, as well as much of northern, eastern, and central Africa. It has a genome consisting of three negative-sense single-stranded linear RNA segments: small (S) (1.7 kb), medium (M) (5.4 kb), and large (L) (12.2 kb).

The natural hosts for CCHF virus are small mammals and livestock, such as cattle, from where the virus can spread to humans either via a *Hyalomma* species tick vector or from contact with infected animal body fluids. The virus can also spread nosocomially, particularly to health care workers from hospitalized patients (3, 4).

In October 2012, the United Kingdom reported a confirmed case of CCHF that was imported into the country (the 2nd such case in the United Kingdom) (5) from Afghanistan by a 38-year-old male patient who had been visiting family in the Samangan province of northwest Afghanistan. Having been symptomatic for ~5 days, the patient flew (via Dubai, United Arab Emirates) from Kabul, Afghanistan, to Glasgow, United Kingdom, and was admitted on 2 October 2012. A serum sample from the patient tested PCR positive for the CCHF virus S segment on 3 October 2012; the patient died in the high-security infectious diseases unit at the Royal Free Hospital, London, United Kingdom, on 6 October 2012 (6).

Viral RNA was extracted from the patient serum sample using an EZ1 DSP virus kit (Qiagen) as part of the Health Protection Agency (HPA) initial emergency diagnostic response. Having been confirmed positive by quantitative reverse transcription PCR (qRT-PCR) targeted at the S segment (7), the complete S segment was sequenced within 48 h. Following this initial confirmation, both the M and L segments were sequenced and the assembled sequence data were submitted to GenBank.

All sequencing was performed using standard Sanger dideoxy sequencing technologies and a 3130xl sequencer (Life Technologies), and contiguous sequences were assembled using DNASTAR SeqMan (v10, Lasergene). In all reactions, viral RNA from the patient serum sample was used as the template and reactions were initiated using the Virology and Pathogenesis Group’s (HPA) collection of CCHFv-specific primers, which were validated against our unique collection of CCHF virus isolates and were designed to provide overlapping reads of the entire segments; this facilitated rapid sequencing to commence immediately following the initial diagnosis.

Since CCHF viruses are highly diverse, this sequence information will ensure that assays remain current and are able to detect circulating strains of the virus, in addition to allowing current phylogenetic and epidemiological studies. This rapid response was, and continues to be, underpinned by several ongoing assay development and surveillance-based projects that ensure the capacity to respond urgently to such cases, as well as to carry out important surveillance programs in endemic areas (8–10).

### Nucleotide sequence accession numbers.

Sequence information for each of the three segments was submitted to GenBank as each was completed, and they have the following accession numbers: for S-Segment, JX908640, for M-Segment, KC344856, and for L-Segment, KC344855.
